# Trends and factors associated with dairy calf early slaughter in Ireland, 2018–2022

**DOI:** 10.3389/fvets.2023.1178279

**Published:** 2023-05-25

**Authors:** Andrew W. Byrne, Stephanie Ronan, Rob Doyle, Martin Blake, Eoin Ryan

**Affiliations:** Department of Agriculture, Food and the Marine (DAFM), Agriculture House, Dublin, Ireland

**Keywords:** sustainable agriculture, dairy youngstock, calf management, neonatal calves, Ireland

## Abstract

Dairy systems require that each cow calves annually to have an efficient milk production cycle. In systems where milk production is maximized, the male offspring from dairy breed sires tend to have poor beef production traits and, therefore, can be of low economic value. Few studies have been published on the factors impacting early slaughtering of calves in peer-reviewed literature. Here we present an analysis of national data on calves slaughtered from 2018 to 2022 in Ireland. Data (Jan 2018-May 2022) on all cattle <6 months of age were collated at a national level and were described at calf-, herd-, and county-levels. These data were statistically analyzed at per-capita slaughter rates (calves/calf born) using negative binomial regression models with an offset. There were 125,260 calves slaughtered early (1.09% of total births) recorded in the dataset from 1,364 birth herds during the study period, of which 94.8% (118,761) were male. 51.7% were classified as Friesian-cross (FRX), 11.5% Friesian (FR) and 32.1% Jersey-cross (JEX). The median age at slaughter was 16 days (Mean: 18.9 days; IQR: 13–22). The median calves/herd slaughtered was 16 (mean: 91.8); median calves/herd/year slaughtered was 21 (mean: 42.0). There was substantial variation in counts of calves slaughtered across herds, years, and counties. Herd calf slaughter rates and per capita calf slaughter rates increased significantly in 2022, with the highest rates over the time series. Calf slaughter rates varied significantly with herd size, year, and major breed (Jersey; JE). Herds which were more recently established tended to have higher calf slaughter rates. Herds that repeatedly slaughtered calves over 2 or more years tended to be larger and slaughtered more calves/herd/year. The slaughtering of calves is not widespread across the dairy industry in Ireland. The distribution of calves slaughtered per herd demonstrate that a small number of herds contributed disproportionately to calf slaughter numbers. Such herds tended to be very large (herd size), more recently established (2016 onwards), and have higher proportions of JE/JEX breed cattle. The outcomes of the present study provide an evidential base for the development of targeted industry-lead interventions with the aim of ending the routine early slaughter of calves.

## 1. Introduction

Dairy systems require that each cow calves annually to have an efficient milk production cycle. The calves produced from the dairy herd have a range of purposes depending on the on the breed of the chosen sire; male and female calves bred from beef breed sires enter the beef production system. When a dairy breed sire is used the female calves are often kept and reared within the dairy herd as replacement dairy animals or sold. However, the male offspring from dairy breed sires tend not to be retained, which in some circumstances, results in their early slaughter. An important point is that, in countries like Ireland, these animals are slaughtered for human consumption, under official controls which ensure that animal welfare is respected, and that this process is a viable economic activity as opposed to systems where calves are slaughtered for disposal which is a cost to those production systems. Notwithstanding this, the slaughter of such male dairy calves is an emerging ethical issue associated with dairy systems in several countries worldwide (1–3). Male dairy calves can sometimes be viewed as a “by-product” of dairy farming, and indeed can be considered economically surplus to needs (4). This problem can be further compounded by the increasing pressure of dairy production, and specialization, including breeding programmes that maximize traits that were beneficial to dairy production (5, 6), but with diminished traits in offspring regarding integrating into beef systems (4). Therefore, male dairy calves, especially those calves that are the progeny of dairy^*^dairy breeding, can be less profitable (and often without any financial value) given input costs (4). While change (in terms of the production of low value dairy calves and their early slaughter) has been called for from several different stakeholder groups and societal actors (e.g., the public, producers), the solutions can present challenges.

Male calves from the dairy herd can be raised for beef production using integrated systems, however, this would require some reversal on breeding goals that sought to maximize milk production gains, which could in turn have impacts on dairy herds efficiency and profitability. Breeding indices have been developed to select for beef bulls that can result in progeny with superior carcass and growth performance, and so increasing calf profitability, while minimizing the collateral effects on cow performance in terms of pregnancy, calving, and milk production (7). Such integrated systems and innovations, in terms of genetic indices, development has coincided with an increased interest in beef-on-dairy production systems in several countries (8, 9).

In several countries, male dairy calves enter veal industries [e.g., (10)]. However, generating supply chains of male dairy calves for veal production locally can be challenging where there is no indigenous/local market demand. Indeed, market demands can also be impacted by consumer sentiments toward slaughter of younger animals, public awareness or product expectations (4, 11). Furthermore, year-round veal production models require consistent supply, which is problematic where there is strong seasonality in calving numbers, such as in countries with primarily pasture-based farming such as Ireland where tight spring calving systems are employed. Reciprocally, the “glut” in calf production during a tight calving period can have impacts in terms of temporal oversupply. Such circumstances can lead to the long-distance transport of male dairy calves to other regions or countries where such markets exist (12). This transportation of calves raises other ethical and welfare considerations (13).

The use of sexed semen (14) is an upstream, and potentially ethically and financially attractive, option for reducing the production of male dairy calves (15, 16). However, sexed semen can suffer from availability, capacity, market, and conception rate challenges, which may make it unattractive to some dairy producers (15).

The slaughter of male dairy calves has become a significant socio-ethical issue among stakeholders, particularly given consumer sentiment and societal views (1, 15, 17–20). In Ireland, the early slaughter of predominantly male dairy calves has been increasing in numbers and has been associated with the change in the milk production sector post the lifting of EU Common Agricultural Policy (CAP) restrictions on production (12, 21). While official statistics on the number of calves slaughtered by sex and age is open access available online (22), there has been limited exploration of the data published to inform policy development. One vehicle for codesigned policy development regarding calf welfare in Ireland is the Calf Stakeholder Forum, a body composed of stakeholders from the Irish dairy industry and the Irish Department of Agriculture, Food and the Marine (DAFM). The forum sought analysis of calf slaughter patterns and trends to inform policy discussions and industry considerations. The present paper has the objective of providing an overview of recent trends in calf slaughter in Ireland and exploring pertinent factors that were associated with the numbers of calves slaughtered on farms during the study period in 2018–2022, with the intention of providing evidence for informed targeted policy development.

## 2. Methods

### 2.1. Data

Data on calves from “dairy” herds, defined as any animals <6mths of age from a herd with a major breed type that was dual purpose or dairy, were obtained from the Animal Identification and Movement System (AIMS) within the Department of Agriculture, Food and the Marine. Major herd-level breed types (and their crosses, signified with an “X” after the code) included Friesian (FR/FRX), Jersey (JE/JEX), Norwegian red (NR), Ayrshire (AY), Meuse Rhine Yssel/Issel (MY), Partenaise (PT), Shorthorn (SH), and Simmental (SI). This resulted in a dataset where 99.8% of herds were FR/FRX (90.0%) or JE/JEX (9.9%) majority breed herds. It should be noted that breed types (and crosses) are those reported by farmers when calves are registered. AIMS provided data on animal birth herd, last herd of residence pre-slaughter, herd size, calves registered per herd, county, health status (testing results from national BVD and bTB programmes during the year that the calf was slaughtered). The latter factors were included to explore whether disease outbreaks within herds, and subsequent restrictions on animal movements, may have explained the slaughter of calves within herds.

### 2.2. Analysis

Firstly, the data were described using descriptive statistics, including trends in slaughter numbers across calendar years, per herd basis, and on a herd/year basis. Data were arranged and managed using MS Excel (23) and Stata 16 MP (24). The data were cleaned and any observations with missing values were omitted from the final dataset before analysis (*n* = 10).

Calf slaughter numbers were modeled at univariable and multivariable levels using a negative binomial distribution, due to the skewed outcome distribution where the variance was larger than the mean (25). A likelihood ratio test was used to assess whether the alpha parameter, the dispersion parameter, was significantly different to zero—which is a test of whether the negative binomial model fitted the data better than a Poisson model (26). The data were modeled in two ways—raw counts of calved slaughtered and calf slaughter rates.

The raw number of calves slaughtered per herd per year were modeled as counts (i.e. without an offset) for spatial and trend analysis. For temporal trend analysis, time [year] was modeled both as continuous predictors to establish the trend (β parameter being negative, positive, non-significant from zero), and as a categorical variable, to establish whether there were interannual variation in the outcome and to establish non-linear trends.

The slaughter rates were modeled using the number of calves born/herd/year as an offset. This was to account for the variable number of calves born per herd per year, and therefore making the model a rate model. A multivariable model was built to explore associations with slaughter rates and year (temporal; categorical), herd size (scaled to per 100 unit increase; continuous), the period/era when the herd was established (registered; <1990; 1990–2003; 2004–2007; 2008–2011; 2012–2015; 2016–2021; categorical), the major breed type (Friesian (inc. crosses), Jersey (inc. crosses) or “other”; categorical), the BVD status (positive or not; binary) and the bTB herd status (positive or not-positive; binary) during the year of the animal's birth. Standard errors were adjusted using the clustered sandwich estimator to allow for intragroup correlation (27), due to some herds being represented more than once within the dataset. No model building strategies were employed when developing the multivariable model, as all selected variables were forced into the model, irrespective of their *p*-values. *Post-hoc* tests were employed to assess differences between categories in categorical predictors, with Bonferroni correction employed where multiple tests were used to avoid potential type I errors.

All models were fitted using Stata 16 MP (24). Rates were mapped at county aggregate level using the open source Geographical Information System QGIS (28).

## 3. Results

### 3.1. Descriptive analysis

The dataset contained information on 125,260 slaughtered calves from January 2018 to May 2022 (therefore, 2022 was a partial calendar year), from 1,364 birth herds. Over the same period there was 11,530,000 births from all cows registered in Ireland, therefore the number of slaughtered calves meeting our inclusion criteria represents 1.09% of the total born (rounded to the nearest 1000; 2022 data provisional; AIMS reports 2021).

Mean herd size of birth herds of slaughtered calves was 352 animals (median: 286; IQR: 183-462; Figure 1A). Mean herd size increased incrementally over each year of the study, with a mean of 317 (median: 263) in 2018 rising to 432 in 2022 (median: 362; Figure 1B).

**Figure 1:**
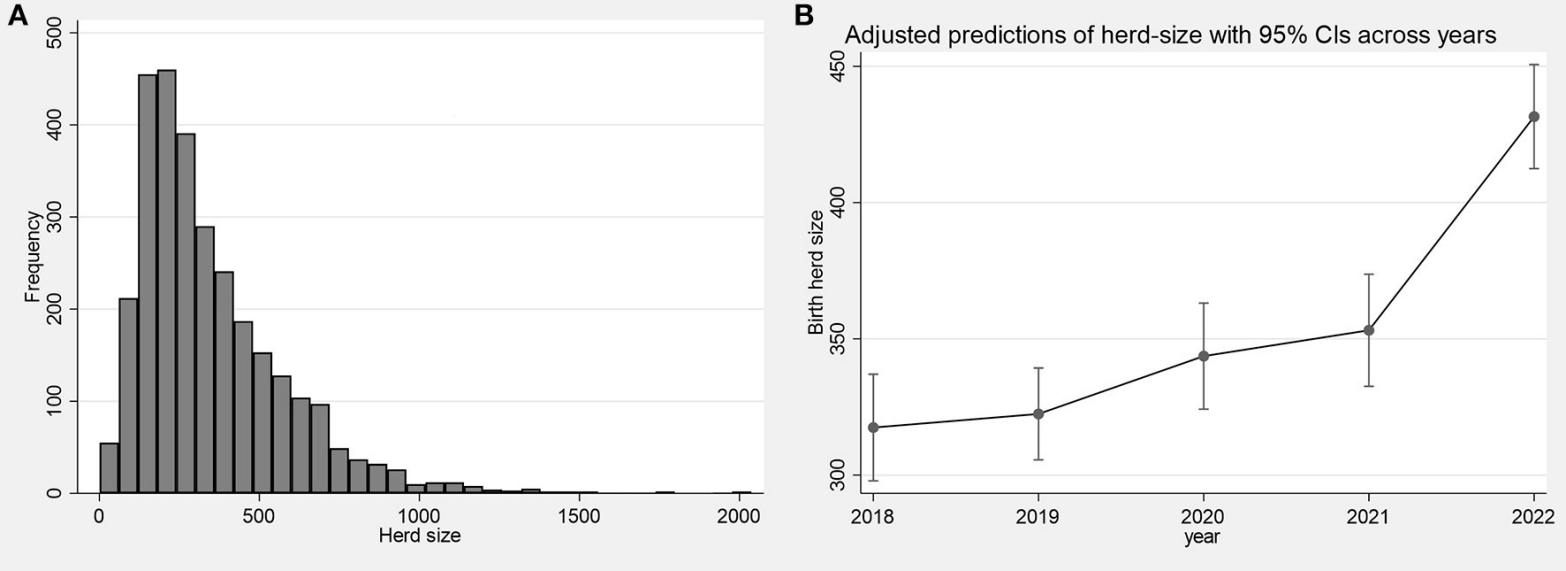
**(A)** Frequency distribution of herd sizes of herds that slaughtered calves during 2018–2022; **(B)** Mean size of birth herds across years of the study period.

Across all the slaughtered calves within our study dataset, 94.8% (118,756) were male. The median age at slaughter was 16 days (Mean 18.9 days; IQR: 13–22 days; 99^th^%ile: 55 days; see Supplementary Figure S1).

The highest number of calves slaughtered occurred in 2019 with 30,144 (24.07% of total), the lowest in 2018 with 16,665 (13.3%). However, only a partial year was represented in 2022 (up until May 2022), and 2022 had the highest calf slaughtering in the months April and May across the time series (Supplementary Table S1). This pattern in 2022 was also reflected in the mean age of calf slaughter in May, which increased from approximately 23.1 days in 2019 to 28.6 days in 2022. There was also an increased number of herds sending calves for slaughter during April and May (Min: 201 herds in 2021 to 348 herds in 2022) and their mean number of calves sent in 2022, relative to other years within the time series (from mean 8.4 (SD: 12.8) in 2018 to 16.5 (SD: 19.4) in 2022; Supplementary Table S2).

Regarding breed, 51.7% of calves were FriesianX, 11.5% Friesian (63.2% total), while 32.1% were recorded as JerseyX. It should be noted that there was a significant decline in the representation of JEX calves slaughtered over time, from a high of 38.8% in 2019 to 23.2% in 2022 (Supplementary Figure S2). At herd level, 73% herds included in the dataset had some Jersey breed (or cross) cattle present (1,003/1,371).

121,576 (99.5% of tested animals) of slaughtered calves were BVD test negative, excluding 3041 calves without a BVD result (2.4% of total); 50 animals were test positive (0.04%); 589 (0.4%) were of unconfirmed status at the time the data were collected. 110,413 (88.1%) of slaughtered calves were born into a herd which did not experience a TB breakdown during the year of its birth.

**Figure 2:**
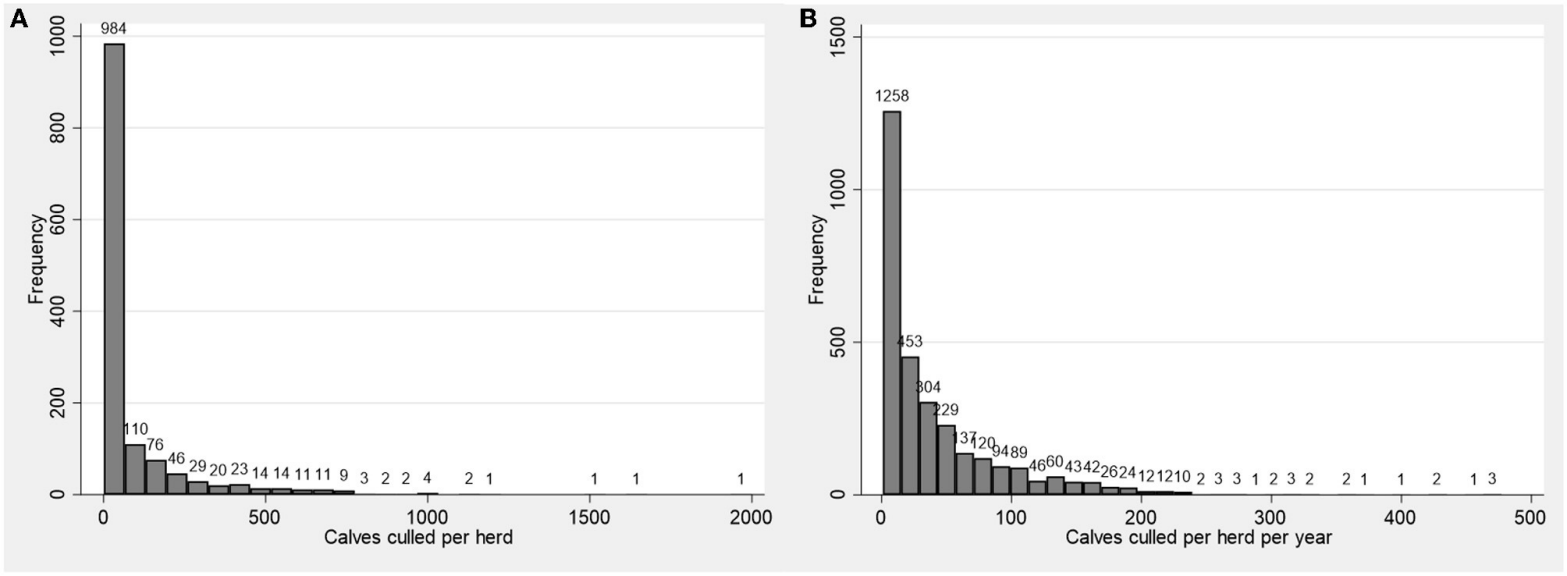
Frequency distribution of calves slaughtered in Ireland between 2018–2022 on a per herd basis **(A)** and a per herd per year basis **(B)**.

The median slaughtered calves per herd was 16 (mean: 91.8; IQR: 2–84); the median calves/herd/year was 21 (mean: 42.0; IQR: 5–56). Both distributions were highly positively skewed, and long-tailed (Figures 2A, B), meaning that there were few herds contributing disproportionately to the number of calves being slaughtered.

Only 14.9% (203/1,364) of herds in the data set slaughtered calves in each year of the time series (5 years), whereas 51.3% of herds slaughtered animals in 1 year of the time series (700/1,364; Table 1). Herds that repeated calf slaughtering over years tended to slaughter on average more calves, for example, herds that only slaughtered for 1 year sent a mean of 10.05 (SD: 17.30) animals, whereas herds that repeatedly slaughtered caves over the 5 years sent a mean of 91.87 (SD: 67.95) to slaughter (negative binomial model; 1-year referent vs.: 2-years: IRR: 2.29; *P* < 0.001; 3-years: IRR: 3.53; *P* < 0.001; 4-years: IRR: 5.29; *P* < 0.001; 5-years: IRR: 8.92; *P* < 0.001). Furthermore, herds that repeated calf slaughtering over years tended to be larger on average, ranging from 255.7 (SD: 201.93) for herds that slaughtered calves for 1 year only to 443.9 (SD: 251.35; Table 1) for herds that slaughtered calves across five calendar years. A linear regression model with a mean herd size (scaled to per 100 animals) outcome, and a categorical variable representing the number of years each herd was represented within the dataset, confirmed that herd sizes significantly increased with more years where calves were slaughtered (1 year referent vs.: 2 years: β: 0.51; *p* = 0.002; 3 years: β: 0.58; *p* = 0.006; 4 years: β: 1.43; *p* < 0.001; 5 years: β: 1.90; *p* < 0.001).

**Table 1:** Herd level classification based on how often dairy calf slaughtering occurred across years 2018–2022 in Ireland.

**No. years where calves were slaughtered**	**No. herds**	**% total**	**Mean herd size (HS)**	**HS Std. Dev**.	**Mean calves slaughtered/ herd/year (MC)**	**MC Std. Dev**.
1	700	51.32	255.70	201.93	10.05	17.30
2	224	16.42	305.22	238.15	23.12	36.58
3	126	9.24	312.99	196.73	35.48	37.40
4	111	8.14	400.37	256.74	50.87	55.64
5	203	14.88	443.87	251.35	91.87	67.95
Total/mean	1,364	100	308.90	230.81	30.04	47.49

HS, Herd size; MC, Mean number of calves.

### 3.2. Trends in calf slaughter

**Figure 3:**
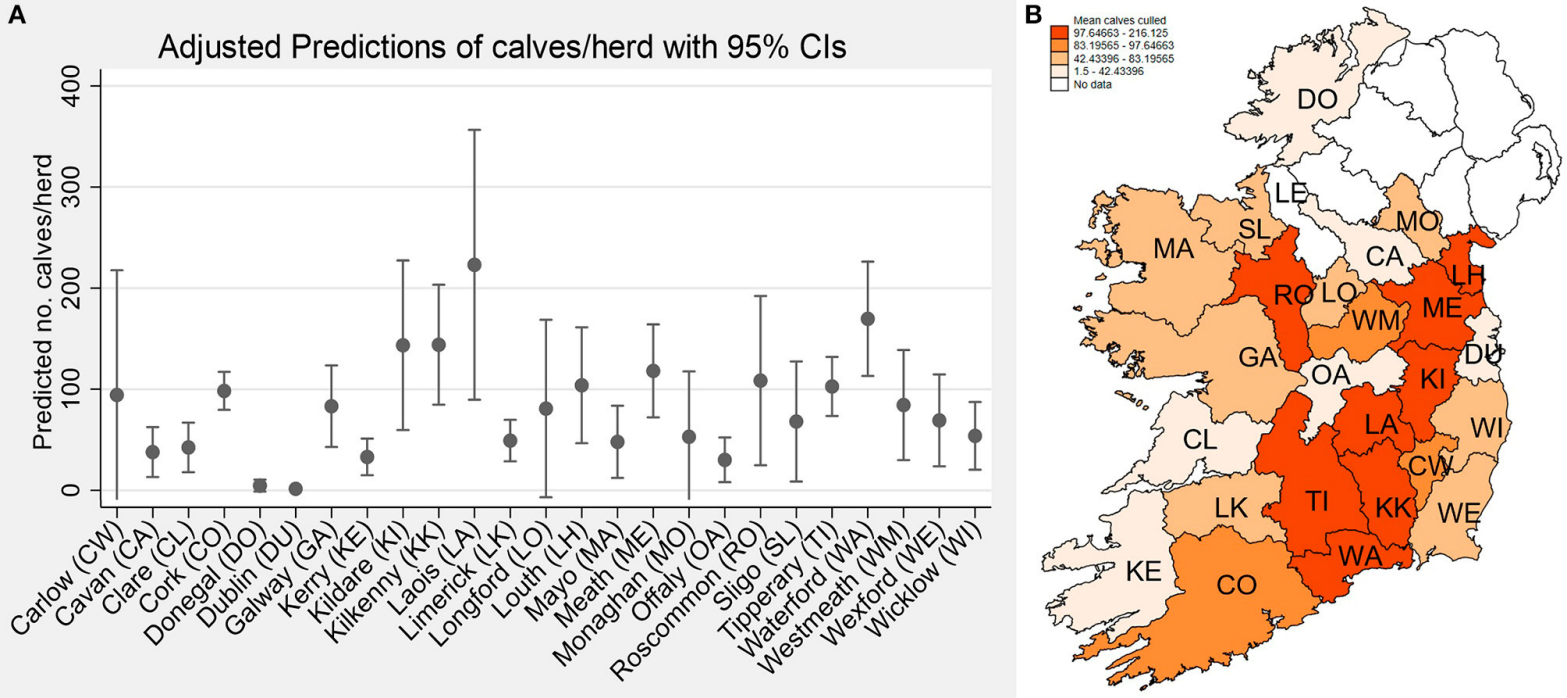
**(A)** Predictions of calf slaughter counts per herd over 2018–2022 across counties of Ireland; **(B)** County level map of variation in slaughter per herd. Counties are presented with their first two letters, with the exception of: LH, Louth; CW, Carlow; WM, Westmeath; KK, Kilkenny.

There were herds that sent calves to slaughter in 25 of the 26 counties in Ireland. Only Co. Leitrim (north midlands; LE on Figure 3) was not represented within the dataset. The highest number of calves slaughtered per county occurred in Co. Cork (*n* = 40,618; 32.4%; 1^st^ rank county dairy cow population), followed by Waterford (*n* = 16,965; 13.5%; 6^th^ rank county dairy cow population), and Tipperary (*n* = 13,867; 11.1%; 2^nd^ rank county dairy cow population). A univariable negative binomial count model suggested that mean calves slaughtered per herd was highest in Counties Laois, Kilkenny, Waterford and Meath (Figures 3A, B).

**Figure 4:**
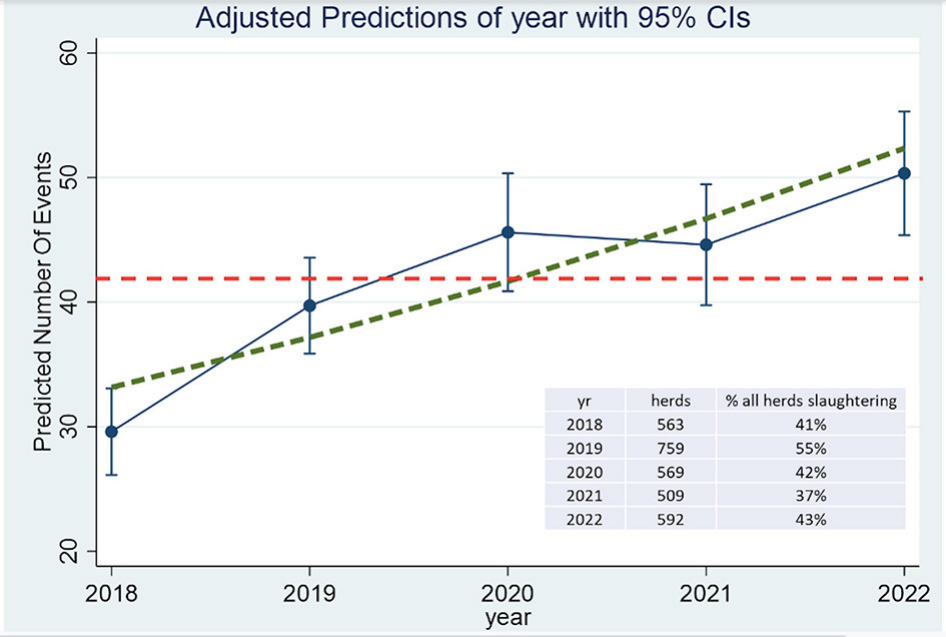
Trends in the predicted mean number of calves slaughtered per herd by calendar year from a negative binomial model. The red line represents the overall mean. Green dashed line is the linear fitted prediction. The blue line presents the predicted yearly mean number of calves slaughtered per herd with 95%CI with year treated as a categorical variable.

The linear trend from a negative binomial model of the counts per herd suggested a mean increase of 112% (incidence rate ratio; *p* < 0.001) per annum (Figure 4). Treating year as a categorical variable suggested that there was a general increase in slaughter numbers per herd with the highest counts in 2022.

### 3.3. Multivariable offset count model

The offset count model is presented in Table 2, where the outcome was calf slaughter rate (per calf born during the calendar year). Herd size was fitted as a linear predictor but scaled so that the parameter estimates relate to a change of 100 animals. The year the farm business was established (when the herd was first registered with the Department of Agriculture, Food and the Marine) was categorized into 6 time periods; (1). the year of establishment was not recorded pre-1990, and therefore formed one block; (2). Fewer herds in the dataset were established from 1990 to 2003, and therefore formed a block; and thereafter 4-year blocks from 2004 onwards with similar numbers of observations. Given the dominance of Jersey and Friesen majority breeds, a three-level categorical predictor was offered to the model, with the final model being all “other” major breeds within herds. This model contained 2985 observations (slaughtered calves per herd year, offset by the number of calves born, with no missing data), from 1364 herds. Overall, the model significantly explained variation in the outcome relative to a null model (Wald χ2(df:14) = 287.36; *p* < 0.001).

**Table 2:** Multivariable negative binomial regression model relating selected factors with the rate of calf slaughtering in dairy herds in Ireland 2018–2022.

**Calf slaughter rate**	**IRR**	**Std. Err**.	***z***	***P*** **> *z***	**Lower 95%ci**	**Upper 95%ci**
Herd size (per 100)	1.075	0.011	7.110	< 0.001	1.054	1.096
**Year**						
2018	Ref.					
2019	1.311	0.050	7.040	< 0.001	1.216	1.414
2020	1.335	0.058	6.670	< 0.001	1.226	1.453
2021	1.257	0.060	4.780	< 0.001	1.144	1.380
2022	1.490	0.073	8.160	< 0.001	1.354	1.639
**Business est**.						
Pre−1990	Ref.					
1990–2003	0.830	0.096	−1.600	0.109	0.661	1.042
2004–2007	0.980	0.138	−0.140	0.887	0.744	1.292
2008–2011	0.871	0.086	−1.390	0.166	0.717	1.059
2012–2015	1.173	0.150	1.250	0.211	0.913	1.508
>2016	1.645	0.136	6.050	< 0.001	1.400	1.934
**Major herd breed**						
Friesen	Ref.					
Jersey	1.704	0.108	8.430	< 0.001	1.506	1.929
Other	0.872	0.169	−0.710	0.480	0.597	1.275
**BVD status**						
Not-positive	Ref.					
Positive	1.155	0.203	0.820	0.411	0.819	1.631
**bTB status**						
Not-positive	Ref.					
Positive	1.122	0.060	2.160	0.031	1.011	1.246
Constant	0.095	0.005	−41.740	< 0.001	0.085	0.106

Offset is the number of calves born.

**Figure 5:**
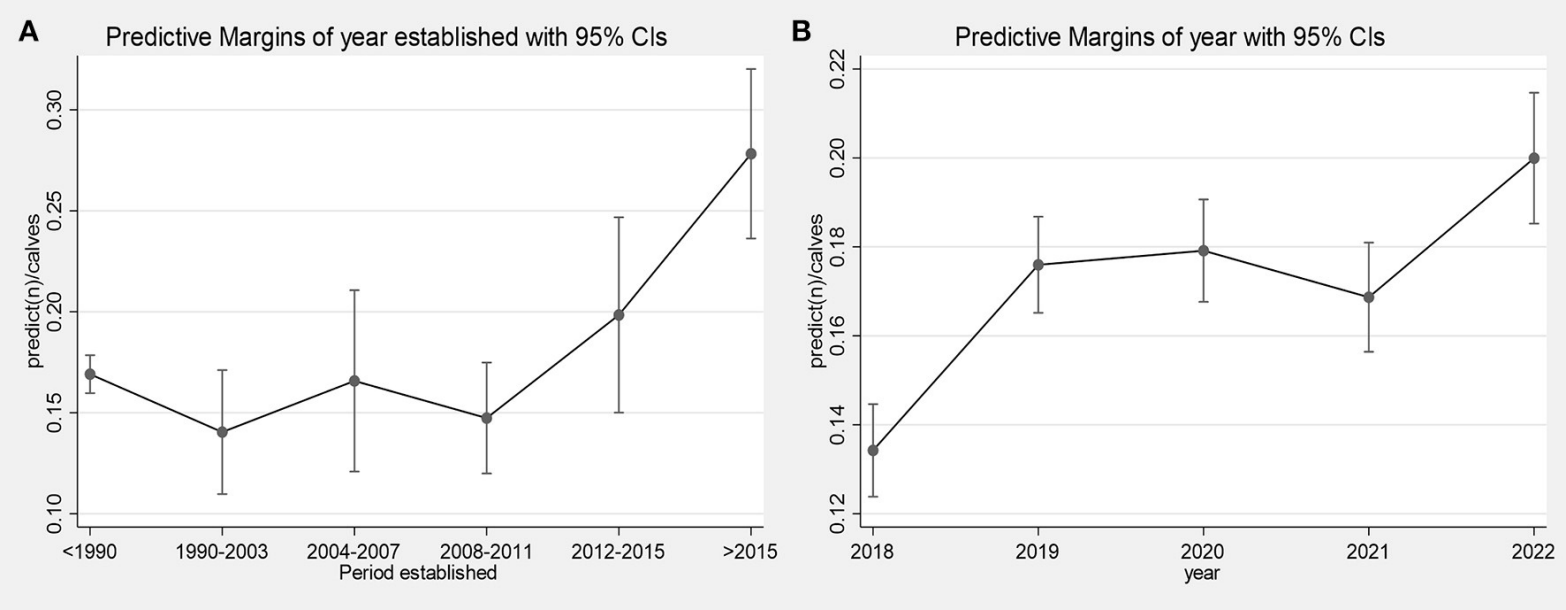
Predicted marginal effect from a multivariable negative binomial model of the slaughter rate (offset: calves born) relative to the period when the herd business was established (bus_yr_cut) modeled as a categorical variable **(A)** and the year **(B)**.

There was a positive trend for larger herds to slaughter more calves (IRR per 100 animals: 1.075; 95%CI: 1.054–1.096), while controlling for covariables, mirroring descriptive results. The slaughter incident rate ratios (IRR) were higher for all years, relative to 2018 (*p* < 0.001), with the highest rate being 2022 (IRR relative to 2018: 1.490; 95%CI: 1.354–1.639; Figure 5). *Post-hoc* Wald tests suggested that 2022 had significantly higher IRRs than years 2019, 2020 or 2021, respectively (χ2: 18.22; DF: 3; *P* < 0.001, with Bonferroni correction for multiple tests). The lowest predicted marginal calf slaughter rate was 0.134 in 2018, rising to 0.200 in 2022 (Figure 5).

The era/period when the herd was established explained significant variation in the outcome (χ^2^: 39.76; DF: 5; *P* < 0.001), however this was primarily driven by a significant increase in slaughter rates for herds established between 2016–2021 (marginal rate: 0.278) in comparison with other eras (e.g. marginal rate was lowest for herds established between 1990–2003 at 0.140). The IRR for herds established post-2015 relative to herds established pre-1990 was 1.645 (95%CI: 1.400–1.934). *Post-hoc* tests suggested herds established between 2016–2021 had significantly higher slaughter rates than herds established in 1990–2003 (χ^2^: 25.00; *p* < 0.001), 2004–2007 (χ^2^: 10.75; *p* = 0.001), 2008–2011 (χ^2^: 26.15; *p* < 0.001), and 2012–2015 (χ^2^: 5.35; *p* = 0.021).

Herds where the majority breed were Jersey or Jersey cross (marginal rate: 0.283) had significantly higher calf slaughter rates relative to herds where the majority breed was Friesian or Friesian cross (marginal rate: 0.166; IRR: 1.704; 95%CI: 1.506–1.929). There was no significant difference in the calf slaughter rates between Friesian/Friesian cross herds, and herds where the major breed was “other” (marginal rate: 0.872; *p* = 0.480).

There was no difference in calf slaughter rates depending on herd's BVD status during the year of the calf's slaughter (*P* = 0.411), but there was a small increased slaughter rate for herds which experienced a bTB breakdown during the year of the calf slaughter (IRR: 1.122; 95%CI: 1.011–1.246). The marginal calf slaughter rate for herds without a bTB breakdown was 0.170 (95%CI: 0.162–0.179), in comparison with a marginal rate of 0.191 (95%CI: 0.172–0.210) for herds that experienced a bTB breakdown.

## 4. Discussion

This study is the first to describe the situation regarding the early slaughter of calves from Irish dairy farms. The data presented suggests that the absolute numbers of calves being slaughtered, and the slaughter rates, were increasing over the study period (2018–2022). However, the study also suggests that the total population of herds that are slaughtering calves is very small, relative to the national dairy herd population. For example, this study found that there were a total of 1,364 herds that engaged in the slaughter of calves at some point over the study period, which represents 8.9% of dairy herds in Ireland [*n* = 15,319; or 1.4% of all herds including non-dairy cattle enterprises, *n* = 97,986 (29)]. Furthermore, only 203 herds engaged in the slaughter of some calves during each year of the timeseries, equating to only 1.3% of dairy herds [0.21% of all herds, *n* = 97,986 (29)]. Even amongst herds where the slaughter of calves had occurred, the majority of such herds were slaughtering very few calves (Figure 2). Small numbers of calves being slaughtered during a given year could occur due to any of several reasons, including business issues, farmer welfare issues or disease outbreaks on farm, including cattle movement restrictions in the case of BVD or bTB. The distribution of the number of calves slaughtered per herd is highly right skewed, demonstrating that there is an even smaller cohort of herds that participate in slaughtering larger numbers of calves on a yearly basis. Such herds tend to be larger herds and repeat the practice over years. This may indicate that these herds are “building in” the slaughter of male dairy calves into their business model, or alternatively, a market failure for these calves has occurred.

Previous research in Ireland has suggested that the issue of “surplus” male dairy calves is a significant concern to dairy (15) and beef (18) farmers in Ireland. The slaughter of male dairy calves is not illegal in Ireland, nor is it an animal welfare issue *per se*, under the expectation that the calves are transported appropriately [albeit with the potential welfare risks and distress associated with road transport; (30)] and slaughtered humanely in accordance with the legislation on animal welfare at time of slaughter (14). However, calves that are slaughtered early, particularly if they are slaughtered within days of being born, cannot be said to have had a *good life* if they have not lived long enough to have positive experiences and to develop a diverse range of normal behaviors (31). Therefore, early calf slaughter it is not a desirable best practice from several different viewpoints, including a socio-ethical, consumer confidence and economic perspectives (2, 14, 19, 20). Factors farmers believe to have influenced the recent increase in excess male dairy calves include the abolition of milk quotas within the EU, the increased profitability of dairy farming compared to other cattle farming models, and guidance on dairy expansion from national farmer advisory services (15).

Options to help reduce the slaughtering of male dairy calves have been proposed, including better integration of dairy-beef systems, the increased use of sexed semen in the dairy industry, increasing transport (e.g. for veal production elsewhere) and improving the markets/prices in the beef sector (4, 14, 15, 18). Dairy-beef integration requires some trade-off in milk producing traits for traits that are preferable to beef systems (7). Beef farmers surveyed in a recent study highlighted the importance of breed, health characteristics and conformation were to beef herdsmen when purchasing calves, and reciprocally the raising of male dairy calves is expected to yield poor profit margins and are at the risk of price volatility and market uncertainty (18). Sexed semen is an ethically sound technical solution, with evidence to suggest that it would be palatable to dairy farmers, though, the uptake of sexed semen in Ireland has been modest in the past due to the perceived additional cost and the reduced conception rate (14). However, several simulation studies of high-input, high-output spring-calving dairy systems in Ireland, found that using sex-sorted semen can provide a significant profit advantages (32, 33), although these advantages can be impacted by market volatility, and reliant on the achieved herd fertility (pregnancy rate) performance, and other factors (34). It should be noted that there has been rapid progress toward increasing the availability of sexed semen in Ireland, with two labs opening in 2021 and 2022, respectively, with capacity to produce ~250K sexed straws/annum. The long-distance transport of calves remains a significant means that dairy herds in Ireland use to manage male dairy calves (14). The primary market is the Netherlands, requiring sea transport. The future ability to utilize this market will be determined by legislation at EU level on welfare of animals on long-haul transport (13), by transport companies (35) and also by local market conditions in the receiving countries.

### 4.1. Factors associated with calf slaughter rates

There was an overall increase in the herd-level slaughter rates over time, as evidenced from the multivariable offset count model presented. This trend was pronounced in 2022 relative to all other years. This trend is despite increasing awareness amongst the dairy cattle industry in Ireland of the reputational risks of this practice, which has been the subject of discussion at the Calf Stakeholder Forum. The analyses from this study provide data to inform future policy developments.

There was a trend for larger herds to slaughter more calves on average, relative to smaller herds within the dataset. It should be noted that the herd size of herds slaughtering any calves is far larger than the national average. For example, the overall average herd size in the present study was 352 cattle, which increased to an average of 443 cattle for herds that sent calves to slaughter for each year of the study. These figures represent 3.4 and 4.3 times the size of the average dairy herd in Ireland (based on a mean herd size of 103 cattle in March 2022). Therefore, it is possible that recent dairy expansion is associated with this phenomenon, though we did not have data available for this study to explore whether the recent change in herd size on farms correlated with greater slaughter numbers. Data on the era during which the dairy herd was first registered with the Department of Agriculture, Food and the Marine (established) revealed that slaughter rates were significantly higher for recent entrants to the industry. Post-2015, when milk quotas were abolished, there was a significant shift on the composition of the national herd and enterprise types (6). It is possible that new entrants may have had less capacity to develop dairy-beef integration partnerships, or possibly new entrants were more focused on milk production when entering the market—the drivers of this finding remains to be seen, and further research is required to understand these patterns.

There was a trend toward herds which experienced a bTB herd breakdown during the calendar year to have higher calf slaughter rates (*p* = 0.03). In Ireland, the disclosure of tuberculin reactors or other evidence of bTB infection on farm leads to restrictions on the movement of cattle off farm (36). The association with increased slaughter rates may indicate that challenges around trade restrictions due to being “locked up”, could have impacted the normal management of calves on the farm. While the Irish bTB eradication programme allows for the movement of calves from bTB-restricted herds to controlled calf rearing units since 2019, uptake of this option has been limited due to the regulatory requirements necessary to manage the disease risk. Herds whose normal business model involves moving male dairy calves off-farm at around 2 weeks of age may not have made provision to keep such calves for longer in case of a bTB restriction. However, only a modest proportion of herd-years were coinciding with bTB breakdowns (22% of calves in the dataset were born in a herd during a year with a TB restriction). Furthermore, there is a strong relationship between herd size and bTB risk in Ireland [e.g., (37)], therefore establishing the causative relationship between bTB breakdowns and slaughter rates is not straightforward.

The raw number of calves slaughtered per county generally correlated with the dairy cow population associated with the respective counties. However, while there was some variation in slaughter rates amongst counties, it is not straightforward to attribute what was driving this variation. Individual herd practices could drive such patterns, but it is notable that generally the lowest slaughter rates tend to occur in counties with lower dairy densities (e.g. Clare, 10 cows/km^2^, Donegal 4.6 cows/km^2^), while higher slaughter rates appear to occur in counties with higher dairy densities (e.g. Waterford 49.9 cows/km^2^, Kilkenny 49.7 cows/km^2^; Figure 3B).

Herds with significant Jersey genetics had higher calf slaughter rates, relative to herds dominated by Friesian or other breed genetics. Jersey and Jersey cross animals have been used for their dairy high production value traits (38), but their male progeny tend to have very little value in terms of beef production and are often not considered for beef production as a consequence (7, 14). The decline in the proportion of calves of JEX breed over time may indicate a move within the industry to reduce the amount of Jersey animals within national dairy herds, potentially reflecting the challenges associated with Jersey male calves (i.e. the calves being of low value, restricted markets) and, potentially, in response to reducing the risk of engaging in the early slaughter of calves.

The data show that a small proportion of herds (15% of total herds slaughtering calves in this study; *n* = 203) repeatedly slaughtered calves over the 5 years of the study, and they tended to slaughter more calves per year and be larger in terms of herd size. These herds are a particular important cohort in terms of targeting industry-led interventions to reduce this practice.

### 4.2. Limitations

The data analyzed here included animals up to the age to 6 months, which is in line with the definition of a calf set out in national and EU legislation. However, it is possible that some factors may vary if the study had of concentrated solely on a younger un-weaned cohort, though the vast majority of the study animals were <2-months old at slaughter.

The metrics for BVD and bTB status were somewhat coarse, being the status of the herd during the year of the animal's slaughter. The mean bTB breakdown duration in Ireland is ~6 months (mean: 178 days), however there are a small proportion of breakdowns that extend beyond 3–4 years (39). It is possible that there were some cases where outbreaks may have begun later in the year, outside of the early season where slaughtering occurs. This would have had the effect of over-estimating the impact of bTB status on calf slaughter.

This study did not explore the specific motivations for farmers to send calves for slaughter, nor the attitudes and perspectives of these farmers on issues such as consumer sentiment, dairy industry reputational risk, ethical questions, or societal views of calf slaughter. While these would be valuable questions to explore, they were beyond the scope of this quantitative data-driven analysis.

## 5. Conclusion

The present study has shown that a small cohort of dairy herds in Ireland partook in the slaughter of calves during 2018–2022, and of these herd, a smaller cohort again have repeatedly engaged in the practice over multiple years. Most herds sent a small number of animals to slaughter per annum, but this distribution was highly skewed, meaning a small number of herds had large contributions to the overall slaughter numbers. Herds that did send calves to slaughter tended to be large herds and had been established/registered recently (since 2016). A factor that may have caused some herds to send calves to slaughter may have related to experiencing bovine TB breakdowns and the associated cattle movement restrictions. Given that the cohort engaged in calf slaughtering is small, targeted engagement by industry and policy development to support alternative solutions could address this issue successfully.

## Data availability statement

The data analyzed in this study is subject to the following licenses/restrictions: Statistical data pertaining to calf slaughter are published and disseminated annually online by the Department of Agriculture, Food and the Marine https://www.gov.ie/en/publication/467e3-cattle-aim/#aim-bovine-statistics-annual-reports; Data available upon request due to restrictions. Requests to access these datasets should be directed to Animalwelfare@agriculture.gov.ie.

## Ethics statement

Ethical review and approval was not required for the animal study because this was a retrospective study of statistical data collected as part of national surveillance efforts. The study utilized records of normal meat inspection processes of animals sent to slaughter for human consumption. No ethical approval was required nor sought as no alteration was required to the normal processing of these animals.

## Author contributions

AB: conceptualization (equal), data curation (lead), formal analysis (lead), methodology (lead), project administration (lead), writing—original draft preparation (lead), and writing—review and editing (equal). SR: conceptualization (supporting) and writing—review and editing (equal). RD: conceptualization (equal), project administration (supporting), and writing—review and editing (equal). MB: writing—review and editing (equal). ER: conceptualization (supporting), project administration (supporting), and writing—review and editing (equal). All authors contributed to the article and approved the submitted version.
