# Supplementary material for: Trends and factors associated with dairy calf early slaughter in Ireland, 2018–2022
Source: Front Vet Sci. 2023 May 25;10:1178279. doi: 10.3389/fvets.2023.1178279 (PMC10248060; doi:10.3389/fvets.2023.1178279)

Supplementary material

Table S1: Calves slaughtered over time in Ireland from dairy herds 2018-2022.

Month	2018	2019	2020	2021	2022	Total
**Jan**	3	55	163	56	83	360
**Feb**	6,567	11,737	8,912	8,143	9,907	45,266
**Mar**	7,945	14,592	13,144	11,617	14,076	61,374
**Apr**	1,482	2,891	3,130	2,561	4,504	14,568
**May**	495	695	432	285	1,227	3,134
**Jun**	7	70	50	11	-	138
**Jul**	16	20	20	6	-	62
**Aug**	50	5	8	10	-	73
**Sep**	31	26	8	3	-	68
**Oct**	53	24	42	4	-	123
**Nov**	12	18	21	5	-	56
**Dec**	4	11	19	4	-	38
**Total**	**16,665**	**30,144**	**25,949**	**22,705**	**29,797**	**125,260**

Table S2: Mean number of calves slaughtered per herd, and the number of herds sending calves, in April and May, 2018-2022. There was a steady increase in the mean number of calves per herd slaughtered (8.4 to 16.4) in April and May, and 2022 had the highest numbers of herds sending animals to slaughter (n=348).

**Year**	**Mean**	**Std. Dev.**	**Number of herds involved**
2018	8.4	12.8	234
2019	10.5	14.1	340
2020	15.0	18.4	238
2021	14.2	17.2	201
2022	16.5	19.4	348
**Mean**	13.0	16.9	272

Fig. S1: Age (in days since birth) distribution of calves slaughtered in Ireland 2018-2022.

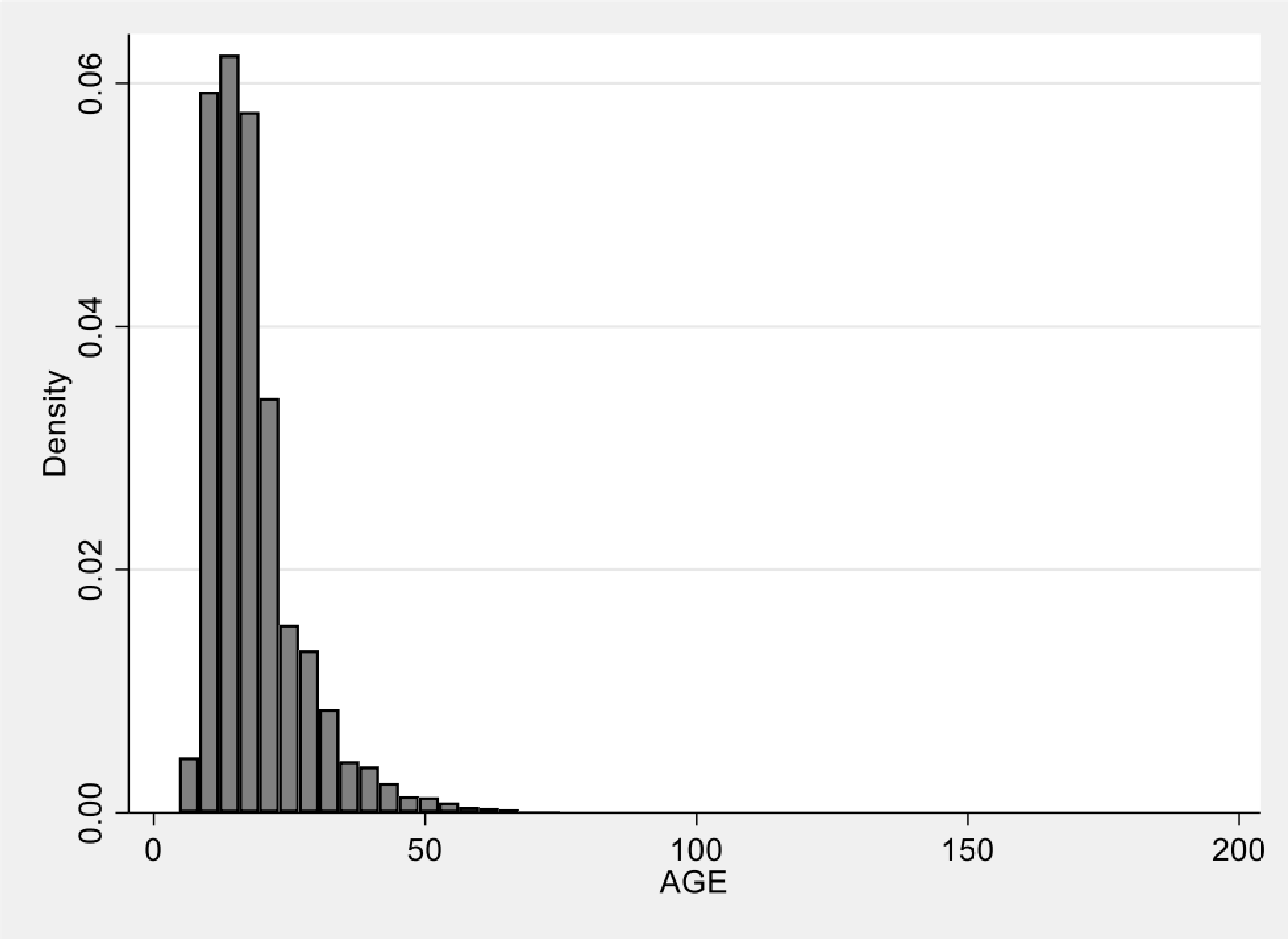

Fig. S2: Proportion of calves that were recorded as JEX per year of the study

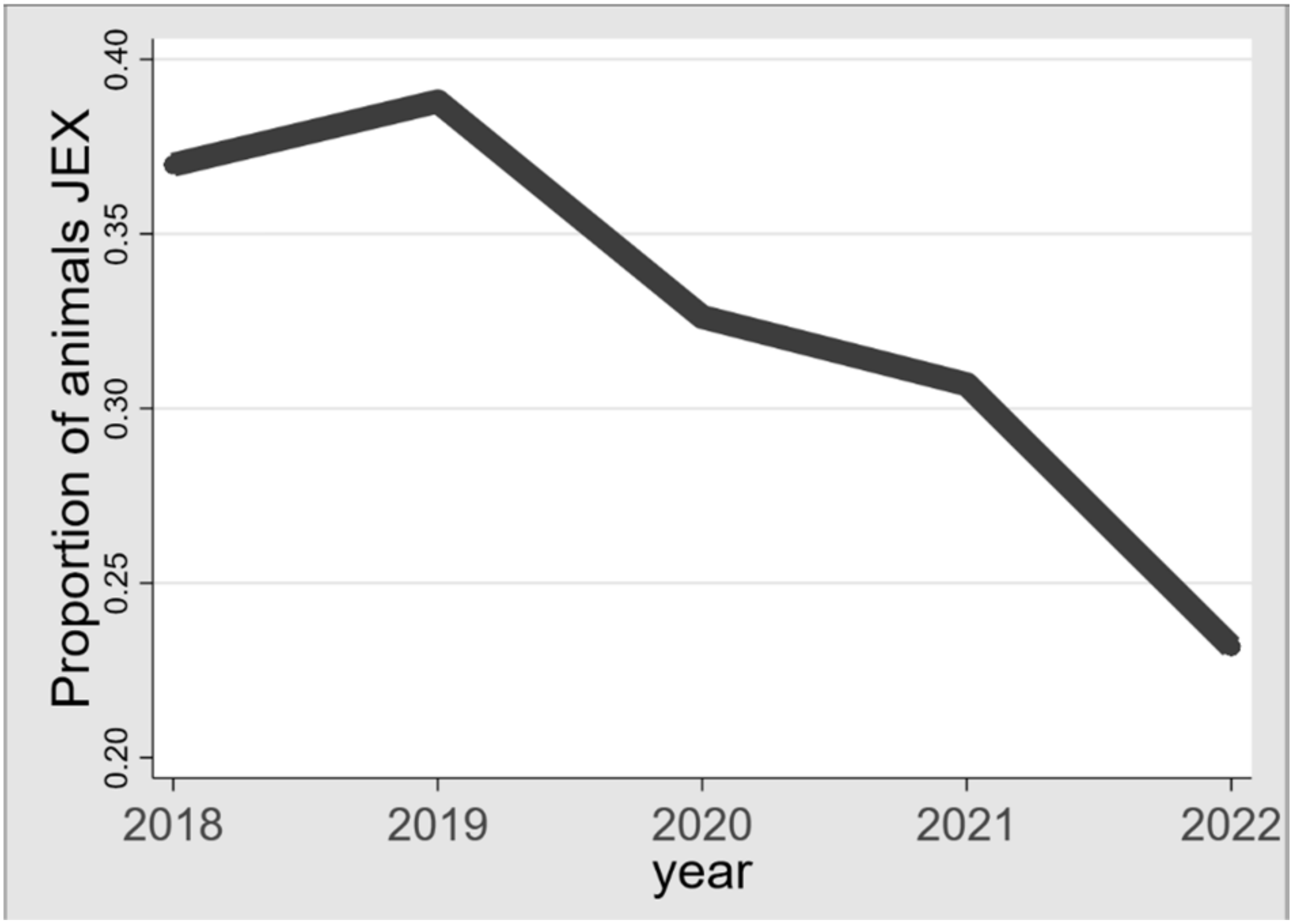

Supplement: Supplementary file 1 [file Data_Sheet_1.pdf]
